# Qualitative Analysis of Attitudes, Knowledge, and Interest in Research of People with Parkinson's Disease and Their Care Partners Receiving Accessible Research Education

**DOI:** 10.1155/2023/5519646

**Published:** 2023-09-11

**Authors:** Maria E. Ramos, Suraj Pothineni, Liang Ni, Allison A. Bay, Todd Prusin, Madeleine E. Hackney

**Affiliations:** ^1^Emory University College of Arts and Sciences, Atlanta, Georgia; ^2^Emory University School of Medicine, Department of Medicine, Division of Geriatrics and Gerontology, Atlanta, Georgia; ^3^Atlanta VA Center for Visual and Neurocognitive Rehabilitation, Atlanta, Georgia; ^4^Emory University School of Medicine, Department of Rehabilitation Medicine, Atlanta, Georgia; ^5^Emory University School of Nursing, Atlanta, Georgia; ^6^Birmingham/Atlanta VA Geriatric Research Education and Clinical Center, Atlanta, Georgia

## Abstract

**Background:**

People with Parkinson's disease (PWP) and their care partners (CP) are underrepresented in research.

**Methods:**

As an eight-week research advocacy training program, TeleDREAMS was designed to increase understanding of, and participation in, clinical research by older adults through topics on the research process. Qualitative analysis was conducted to explore themes from 365 thirty-minute semistructured phone interviews with 32 PWP and 17 CP TeleDREAMS participants. Interviews gauged progress, motivation, and information retention after each weekly module.

**Results:**

Eight salient themes were identified from the interviews, including Understanding the Importance of Advocacy and Becoming Cognizant of Past Advocacy Experiences.

**Conclusions:**

While some findings aligned with weekly module topics, others, such as stated learning preferences and knowledge acquisition of older adults in an educational program, were unexpected. TeleDREAMS may increase interest in community engagement, research participation, and advocacy roles in marginalized and underrepresented participants.

## 1. Introduction

Individuals from racial and ethnic minority groups and those with low incomes are both underrecruited and underrepresented in research studies [1]. Many older adults with Parkinson's disease (PD) and their care partners (CP) are from socioeconomically disadvantaged populations, rural populations, and certain racial and ethnic groups [2]. PD is a neurodegenerative disorder that is progressive in nature and characterized by motor and nonmotor symptoms; symptom range, detection, and rate of progression vary for individuals [3]. Successful methods for engaging underserved research participants include fostering trust and creating lasting partnerships through training health ambassadors from the community [4]. Retaining individuals of health disparate populations presents many challenges, but adaptable and measured recruitment efforts may ensure retention of underserved participants [5].

TeleDREAMS was an eight-week educational telehealth program for adults with Parkinson's disease (PWP) and their care partners (CP) that sought to educate older adults on the research process and role of participation in research. The research advocacy program included participants from diverse backgrounds and attempted to increase research participation among critically underrepresented PWP and CP in research. TeleDREAMS built on the previous two-part, in-person DREAMS Program [6, 7]. TeleDREAMS, however, was implemented via distance-learning, where weekly phone call interviews gauged progress, motivation, and information retention in TeleDREAMS participants.

This study examined the effects of the eight TeleDREAMS educational modules on beliefs and attitudes towards clinical research, the research process, and participation in research in older adults with PD and their care partners. This was accomplished through a qualitative thematic analysis of weekly phone call interviews conducted over the course of the program's eight-week duration. By analyzing these calls, we aimed to learn what participants believed they had learned, what they found most interesting and novel, and what information from the program would be most useful to them. Importantly, we also intended to determine which topics participants were already familiar with, upon which supplemental materials they relied, and what topics should also have been included. We anticipated varied responses and levels of understanding among program participants regarding what they learned from the modules.

Beyond finding participant responses closely aligned with interview questions, we expected to see an increase in knowledge of clinical research opportunities and research processes, an increase in the willingness to participate in clinical research, and an increase in positive attitudes toward research and participation in research. Moreover, we expected these core ideas would evolve to become increasingly apparent in participant responses. We predicted a difference in responses between PWP and CP that would be related to their distinct roles in their caregiving partnership.

## 2. Methods

Emory University Institutional Review Board reviewed and approved the TeleDREAMS protocol (IRB 80676); all subjects provided informed consent before participating in this study and provided consent for publication of the findings. This study has a qualitative phenomenological design. This work is based partially upon a thesis successfully defended by the first author [8].

### 2.1. Participant Recruitment

With help from previous partnerships from DREAMS [9] and patient stakeholder advisors, 51 adults who were either people with Parkinson's disease (PWP) or care partners (CP) of people with Parkinson's were recruited and enrolled for the TeleDREAMS study (Table 1). Strong efforts were made to recruit individuals from historically underserved backgrounds that included ethnic minorities and individuals with low health literacy. Participants were recruited at local community centers and events, within community outreach programs at Emory's Center of Health and Aging, at senior events and educational meetings, at local churches, and at Parkinson's community events.

Participants for the study were selected as part of a convenience sample, as was done in the parent study, Developing a Research Participation Enhancement and Advocacy Training Program for Diverse Seniors (DREAMS) program. For more information about the sample size selection for this study, refer to Schindler et al.'s study [9].

### 2.2. Participants

Participants with PD and CP participants were both recruited throughout the study recruitment process.

CPs were selected for inclusion in this study because of their integral role in PWP care [10, 11]. For example, CPs may be more likely to address important changes in PWP symptoms with their doctors than with PWP themselves [12]. The following inclusion and exclusion criteria were observed for all participants.

A board-certified movement disorders neurologist had provided eligible PD participants with a PD diagnosis prior to study participation. All PD participants came with diagnoses of idiopathic “definite” PD [13] and had had PD one or more years prior to study participation. All participants therefore had met the following criteria at the time of their diagnosis: unilateral onset, exhibiting 3 of the 4 cardinal signs of PD (rigidity, tremor, bradykinesia, and postural instability), and response to antiparkinsonian medication. They did not have familial or young onset PD and were older than 40 at the time of their diagnosis. To be included, all PD participants had to have no other major neurological disorders. For care partners, the inclusion criterium was familial or friendly relationships with a person with PD. Further, care partners had to report they provided “regular” and “ongoing” care of a person with PD, given their roles as spouse, or emotional partner for someone with PD. Paid care partners were ineligible for the study. The amount of care required by different study participants with PD depended on their level of independence. Some care partners were responsible for providing more support for activities of daily living (ADLs) than others.

All participants were expected to speak, read, and comprehend English and to participate in a weekly, thirty-minute phone call interview with the study team. Distance-learning and one-on-one accountability through telephone interviews allowed the participation of rural and mobility-limited individuals. Free transportation to in-person pre- and posttest assessments was provided to decrease some barriers to participation.

### 2.3. Overview of the TeleDREAMS Program

An educational binder was distributed to TeleDREAMS program participants that contained eight separate weekly modules. Participants were expected to independently read the corresponding module for each week. To ensure accessibility for all participants, the weekly modules were approximately 20–30 pages long and were written at an eighth-grade reading level. Also, optional supplemental videos and related web-based resources were provided in footnotes with which participants could engage. The educational material contained modules on understanding clinical research, health topics of relevance to PWP, and health disparities (Table 2).

### 2.4. Data Collection

Participants were characterized for clinical and demographic information with standard health questionnaires administered immediately prior to joining the program at an in-person assessment. The composite physical function index (CPF) was administered to all participants. CPF questions request a self-evaluation of physical and functional ability. Lower scores indicate a person is at greater risk of a loss of function [14]. At this assessment, participants were oriented to the program, given their educational binder, explained how to use the educational binder and informed about weekly calls they would receive from research assistants. Data were collected from 30-minute phone calls with 32 PWP and 17 CP that occurred at the end of each weekly module to ascertain progress and discuss each completed lesson. Research assistants trained in interviewing for TeleDREAMS performed the telephone calls. The questions asked in the interviews (Appendix) mirrored those used for the original DREAMS Program small group discussions [9]. Participants were asked if they read the module, what they learned, what content they found interesting or new, what content they might be able to use in their daily lives, about what topics they might have previously known, and on which supplemental materials they relied. Participants were also asked for suggestions on what content they believed should have been included.

### 2.5. Data Analysis

Staff took field notes during each weekly phone call and analyzed using NVivo 12 and NVivo (Release 1.3.2) software. Data were coded by the first author using both deductive and inductive coding techniques; the calls were thematically analyzed, and key themes were identified. Themes were verified and reviewed by the second and the senior author and consensus was derived using standardized, published methods [15]. The eight salient themes that were most inclusive of participant responses have been considered and identified.

## 3. Results

### 3.1. Participant Characteristics

A subsequent version of this research advocacy training program was designed so that participants received audio recordings of the educational content, in addition to physical binders containing printed text. Though 19 CPs initially enrolled in TeleDREAMS, two CPs were excluded from the sample (resulting in *n* = 17) because the two excluded CPs received weekly content in this new method–as both text and audio recordings. Given that these participants did not receive the material in the same manner, we judged it best for the study's findings to not include their data.

As indicated in Table 1, PWP average age was *M* = 68.06, SD = 8.3, and 69% male; while CP average age was *M* = 66.68, SD = 6.4, and 32% male. Higher composite physical function scores represent less risk for loss of function and are recorded out of 24 points. On average, composite physical function was higher for CP (*M* = 23.06 and SD = 1.4) compared to PWP (*M* = 17.88 and SF = 5.9). The total sample size for this study contained 55% male participants. Both PWP and CP had additional comorbidities that included high blood pressure, heart problems, diabetes, depression, arthritis, asthma, osteoporosis, cancer, cerebrovascular accidents, joint replacements, and vertigo.

### 3.2. Qualitative Findings

**Figure 1:**
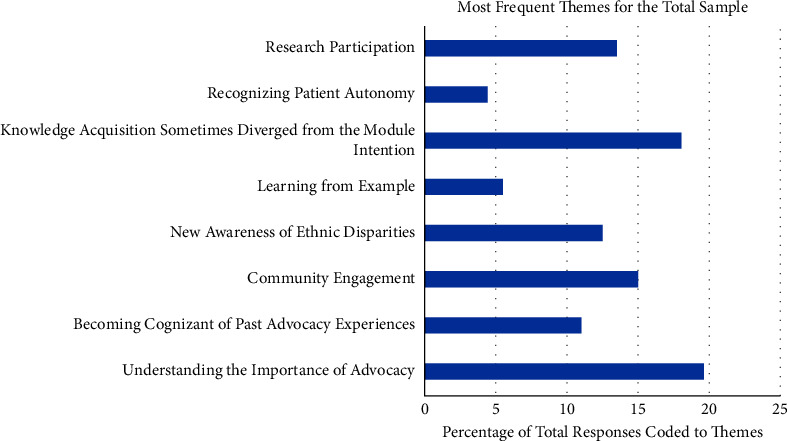
This histogram represents the percentage of 199 total responses that were coded and analyzed to each of the 8 most salient themes. Data resulted from a total of 365, 30 minute phone call interviews placed by staff and conducted every week over the 8 week program for all participants in the sample, including PWP (*n* = 32) and CP (*n* = 17).

**Figure 2:**
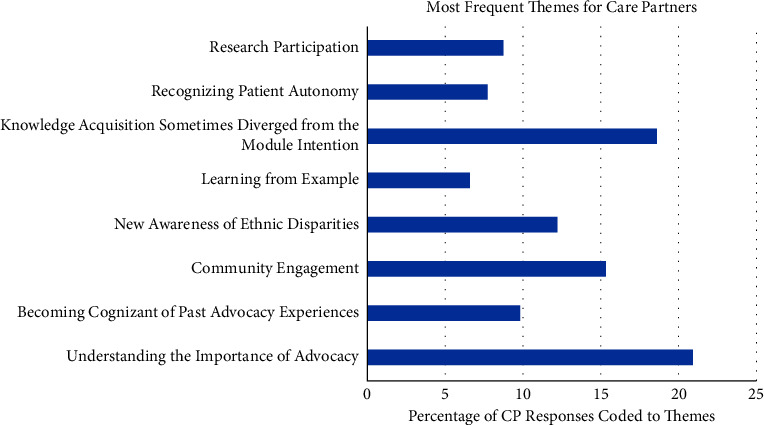
This histogram represents the percentage of 91 CP responses that were coded and analyzed to each of the 8 most salient themes. Data resulted from a total of 122, thirty-minute phone call interviews placed by staff and conducted every week over the eight-week program for CP in the sample (*n* = 17).

**Figure 3:**
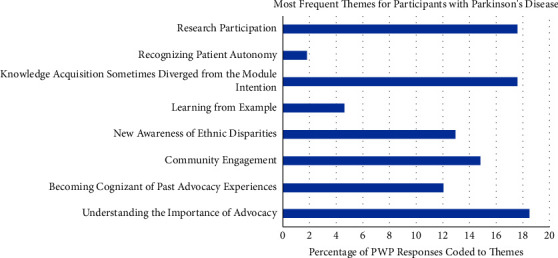
This histogram represents the percentages of 108 PWP responses that were coded and analyzed to the eight most salient themes. Data resulted from a total of 243, thirty-minute phone call interviews placed by staff and conducted every week over the eight-week program for PWP in the sample (*n* = 32).

**Figure 4:**
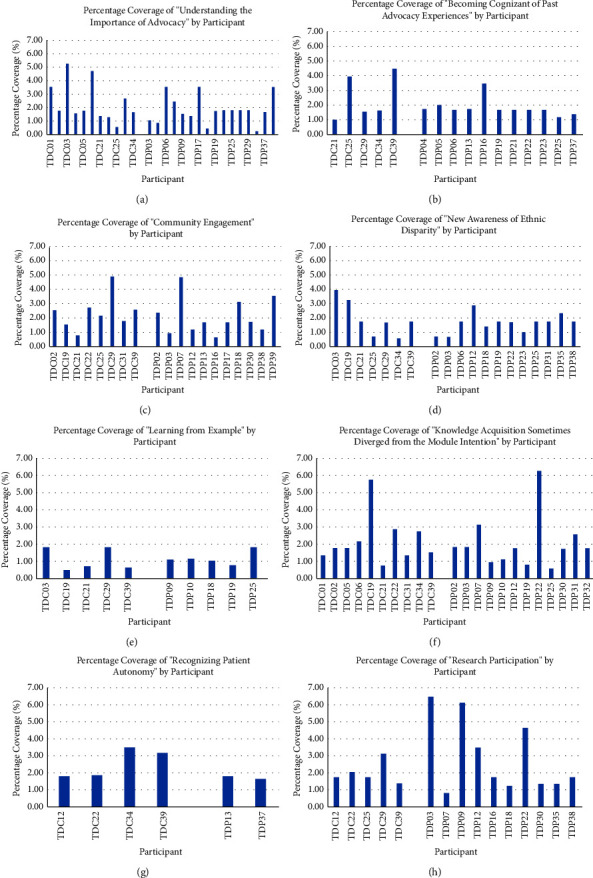
Histograms (a)–(h) of percentage coverage of each of the eight most salient themes. These figures represent the percent coverage of each theme by individual participants. TDC bars indicate CP participants, while TDP bars indicate PWP participants.

The eight most salient themes extracted from CP and PWP responses were as follows: (1) Understanding the Importance of Advocacy, (2) Becoming Cognizant of Past Advocacy Experiences, (3) Community Engagement, (4) New Awareness of Ethnic Disparity, (5) Learning from Example, (6) Knowledge Acquisition Sometimes Diverged from the Module Intention, (7) Recognizing Patient Autonomy, and (8) Research Participation. The percentage of total, PWP, and CP responses that were coded and analyzed to each of the eight most salient themes are shown in detail in Figures 1–3. Additionally, the percentage of time each theme was covered by participant has been produced (Figure 4).

### 3.3. Theme 1: Understanding the Importance of Advocacy

Throughout the eight-week program, participants appeared to understand the importance, universal role, and widespread responsibilities of advocates in research. Some participants were unaware that members of their communities, including themselves, could serve as advocates, though they were able to recognize celebrity advocates. Upon participation in TeleDREAMS, a CP responded that the CP “didn't know that you needed everyday advocates,” while a CP said, “outside of just celebrities, it (advocacy) can be a community effort.” The responsibilities of advocates were detailed through different actions. A PWP described advocates as people who “stand up and make their voices heard” and went on to say “advocates come from different point (s) of view, especially between patient and caregiver.” Lastly, a PWP learned “how important the advocacy program is to get people included in research and clinical trials and how a person like me can be an advocate and help facilitate that.”

Participants also noted the long-term effects of advocacy in healthcare. A CP reported, “…advocacy can diminish the communication gap between participants and researchers” and that “advocacy can help to bridge the gap of underserved populations in research and healthcare.” A CP extended advocacy to a broader scale, saying “An advocate can come in so many different forms. Anyone can become an advocate. If you support something or someone, you can become an advocate.”

Still, a PWP identified a unique difficulty with becoming an advocate, stating, “Being an advocate for other people looks like a good thing. I try to do that in my community. With PD, it is hard to get motivated. So many people withdraw, but even though you have PD, you have to keep going.” A PWP expressed his fear of inadequacy because of health concerns to be an effective PD advocate, saying “I'm worried I'm not a good advocate because I don't speak or remember well.”

Overall, these responses demonstrate that learning about advocacy encouraged some participants to pursue advocacy. A CP said,

“I am excited about advocating for myself and people you meet to get others involved in research and research advocacy, because that's what my wife and I do and will continue to do. It gives us hope that people are dedicated to get others involved in research.”

A CP looked to future steps saying, “I need to learn more about PD and more of the scientific part of PD if I am going to be an effective advocate. I am a retired attorney so being an advocate isn't new to me. I understand some of the thing's advocates can do, but you have to be well informed to be effective.”

### 3.4. Theme 2: Becoming Cognizant of Past Advocacy Experiences

Over the course of eight weeks, participants not only understood the foundations of advocacy but also recognized their own advocacy experiences. Once provided with the formal definition of advocacy, many participants realized they had unknowingly participated in advocacy during their lifetimes, oftentimes mentioning support groups. A PWP said, “I realized I was doing things and didn't realize it. Hopefully things I have done in the past have helped people come into research.” A PWP saw this moment of realization as encouragement for future participation when the PWP said, “It just makes me excited to continue to participate. And to know, I've been an advocate all along but didn't realize that.”

The educational modules brought to mind similarly encouraging experiences for other participants. A PWP revealed decades of advocacy, saying,

“Going through this course has made me aware of all that I have done and continue to do…I have been an advocate for at least eight years.”

A CP even recognized that the CP's spouse had previously engaged in advocacy efforts, saying,

“My husband, in his support group, brought in a speaker, who is a yoga instructor. The yoga instructor talked about how yoga can be therapeutic for those with PD. This is one-way advocates can help the community. Bringing resources to their community and among those resources is research.”

### 3.5. Theme 3: Community Engagement

Aside from research participation, many participants expressed interest in more fully engaging with their communities. The educational modules prompted participants to contribute to preexisting community programs. A CP described how she could now “contribute to the conversations,” of her cancer support group and how she “will share this information with (her) peers”.

A CP even planned on trying dance lessons with her spouse, saying, “dancing has a positive effect on people with PD and stroke, spinal cord injury, so me and my husband are talking about dancing lessons.” A 5PWP shared his engagement with his religious community, stating, “I talked to my church group a little bit and tried to explain to them, and they enjoyed it.”

Some participants expressed interest in leading new programs for their communities, with fitness groups being a popular option. A PWP hoped to create “some kind of studying and exercise at local recreation places that would sponsor those kinds of events,” while a CP detailed previous plans, saying, “I am going to help start a PD boxing group in different parts of Mississippi. I have a meeting with prospects tomorrow.” A CP compared PD care in the United States to PD care in the South American country Colombia, saying, “There is research, clinical trials, and support groups in the US. There is everything here, but nothing in Colombia. Everyone with PD goes to the same doctor because he is the only physician that specializes in it.” This CP hoped to bring change to their Colombian community, saying, “I would like to collaborate with researchers and organizations because people with PD are abandoned there (Colombia).”

The desire to engage with local communities was not shared by all participants. A CP said he was “pulling back from doing stuff” and “looking for ways to be less involved,” but he “might move in that direction again.” Interestingly, learning about the historical underrepresentation of older adults in research led one patient to recognize his own lack of engagement, which he then perceived as a flaw. The CP said, “old people are excluded for convenience's sake. Older people can isolate themselves, which is kind of what I'm doing, which isn't a good thing.”

### 3.6. Theme 4: New Awareness of Ethnic Disparity

While most participants seemed to understand existing ethnic disparities in healthcare and research, those who were unaware of the disparities often expressed a degree of shock after completing related modules. A CP recalled, “I was surprised about the gaps with Latinos and African Americans in research. I did not realize that a larger percentage of Hispanics die from cancer, but few are in the cancer studies.” Other participants felt compelled to make a change in these “dismal results” through outreach efforts. A PWP said,

“I would like to help with the strategy to help overcome that obstacle (participation in research) and get people in those hard-to-find segments…the minorities for example, and the groups that are underrepresented. I think that is a big deal! We shouldn't have those kinds of issues.”

While a CP recognized that she was not from an underrepresented demographic, she still felt “there are areas that (she) probably can reach out to populations that are somewhat diverse, and you can always find people to help translate.” A appreciated the inclusion of ethnic disparities in the module, saying,

“Diversity matters a lot to me, and I am so happy that a section was dedicated to it.”

### 3.7. Theme 5: Learning from Example

Participants seemed to benefit the most from the example-based pedagogical approach of TeleDREAMS. Personal stories and example-based helped participants relate to the educational content and often allowed them to draw similarities to their own lives. A CP said,

“I enjoyed the example of advocates sharing their opinions and giving a more specialized opinion to the research team. They have first-hand knowledge and can contribute to the research. I liked the personal touch of listening to the advocates and their personal experiences.”

Many other participants expressed how they enjoyed the example stories and experiences, with a CP stating, “There were also a lot of interesting statements and testimonials from other advocates. I enjoyed reading them.” Another participant, a CP, followed along the same line and said, “I like the examples. It was very interesting to hear about the research they are doing in other parts.” A PWP found these personal stories “useful to pass along,” perhaps signaling his decision to implement the personal stories into his own discussions about PD and research. Even for a PWP who struggled with cognitive impairment and was unable to recall the examples in detail, the presence of personal stories in the weekly modules still had a profound impact. This PWP said, “it gives real life examples of the types of studies and so forth, and…there's something related to having a real-life example, but my memory is so bad, I'm not sure it will help me.”

### 3.8. Theme 6: Knowledge Acquisition Sometimes Diverged from the Module Intention

Each week, participants engaged with a different module that centered around a new topic related to advocacy, research participation, and the research process. Though each week had its own unique learning objectives, participants acquired different knowledge about the same topics. We observed that what participants learned sometimes differed from the intended goals.

#### 3.8.1. Weeks 1–3

Responses from the first three weeks concerning advocacy, research “nuts and bolts”, and ethics largely suggest that participants acquired knowledge congruent with each module's goals. Quotes from the first three weeks include

From week 1, Advocacy: “I learned what advocacy is and to understand what PD advocates do in research and the different types of projects and possible things ones could get involved with.” (CP)

From week 2, Research Nuts and Bolts: “I like it when you're actually monitoring a specific group of people. I enjoyed learning about the research.” (PWP)

From week 3, Ethics: “I knew there are a lot of ethical practices in place to prevent unethical studies.” (CP)

From week 3, Ethics: “The ethical issues were powerful stories…They knocked me off my chair.” (PWP)

#### 3.8.2. Week 4: Understanding and Interpreting Clinical Trials for Patient Advocates

During the fourth week, focused on interpreting clinical trials, participants appeared to not only understand clinical research, but also an appreciation for learning about how to read research papers. A PWP said,

“(learning) how to read a research paper was interesting, because most people will just read the snapshot before they read the paper. and I thought it was an excellent way to read it…to read the introduction and pick the key questions in your mind and see if you can see any bias in it…Who pays for their research is the question I have! The statistics show…so and so…but who pays to show that research?”

#### 3.8.3. Week 5: Aging and Clinical Research

Most participants were able to relate week five's topic on aging to their own personal experiences with aging. A PWP said that she

“learned that everyone goes through losing some of their abilities to see and hear as they get older and I always thought I could just go to a doctor to get those fixed but might just have to be happy with the way it is.”

Others were surprised at the definition of age discrimination. A PWP, said he “didn't know ageism is a word,” while a CP said, “I never thought about age discrimination and awareness. Never thought about age discrimination but now I know to be aware of it and pay attention about that.” A PWP appreciated information about exercising related to aging, saying,

“The benefits of exercise stuck out to me as well as the benefits of mental exercise. Because I have Parkinson's it is important to see how fast I can do calculations like 143 minus 7.”

#### 3.8.4. Week 6: Informed Consent and Health Literacy

Most participants appear to have gained an understanding of health literacy by the end of the week. A CP said that health literacy “feels like it helps people make appropriate decisions about healthcare based on information.” A PWP noted,

“I learned that I thought I had good health literacy, and I hope I do. After reading the definitions I kind of questioned myself. Especially when I read that small percent of people have good literacy. You had to understand a lot of insurance to qualify as good. Maybe I'm only adequate.”

The other goal for the module was related to informed consent. A PWP said, “I found the information about the consent laws in GA very informative, i.e., directives and such.” Another participant seemed to grasp the importance of informed consent. This PWP said, “For someone who has a minimal amount of education, I can see how it would be very difficult. I would be concerned if it was someone in my family and the doctor would just gloss over the informed consent process.”

Participants reported learning specifically about health insurance models and advanced directives more frequently than health literacy or the broader topic of informed consent. This is an example of a surprising finding that differed from the module's intended goal. A CP, noted,

“I learned about health care systems around the world and how the US is more like a hybrid system. It's more like a a-la-carte. I didn't think about how Medicare and veterans and the different patients access different types of systems instead of one system like in other countries.”

Another participant, a CP, reflected this sentiment when she said,

“I didn't know what the different models of health care were called, and the US is totally out of sync with everyone and that was interesting to learn. I did know that they spend almost 2x as much on health care…I did know the Medicare stuff because I have to go on a supplement policy this year.”

#### 3.8.5. Week 7: Advocacy in Clinical Research

Though expectations of the seventh week were focused on understanding advocacy within clinical research, it appears that participants were intrigued by the collaborative role an advocate can play in research. For instance, a PWP described this, “collaborative aspect of the research,” saying,

“I don't know why it was new, because I've done research before. I still think that reading this it feels more like a collaboration of equals. We need them (researchers), they need us (patients), as opposed to the researchers having all the knowledge and power.”

#### 3.8.6. Week 8: Recognizing Diverse Communities and Becoming an Advocate

In week 8, when learning more about advocacy and recognizing diverse communities in research, most participants took away an understanding of barriers to research. Overall, participants used the term “barrier” in the context of research participant limitations, eleven different times. A PWP said, “I am trying to figure out how barriers apply to the PD community I am in.”

### 3.9. Theme 7: Recognizing Patient Autonomy

Though not explicitly discussed in the weekly educational modules, recognizing patient autonomy was identified in the personal stories of autonomy and the desires of care partners to promote the autonomy of care recipients. A CP, revealed that her participation in research studies has led her to, “want others to realize they have a voice when others feel that they don't.” A CP said, “people need ownership of their healthcare. You have the responsibility to take care of your health.” This same participant illustrated how he, “(has) ownership,” in his decision to use a spreadsheet to track his medical history that he presents to his healthcare professionals as a supplement to his patient charts. A CP recalled instances, where she recognized her husband's autonomy, saying, “You don't need to let everything slide. Sometimes you need to let him (her husband) deal with people himself.” Field notes reveal a PWP found learning about health insurance, “was interesting because she (participant) could relate to insurance. She usually let someone else do it for her, so it was new for her to learn.”

### 3.10. Theme 8: Research Participation

TeleDREAMS modules appear to have fostered interest in scientific research topics among program participants. A CP felt it was her “social responsibility to help find a cure for Parkinson's disease,” while other topics of interest included genetic research and cerebrospinal fluid analysis. Beyond interest in research topics, participants conveyed skills that they gained from the program that benefit their participation in research. For instance, a PWP recalled, “I was actually interviewing for a clinical trial and what they are doing makes a lot more sense. I am learning a lot more than I am able to explain.” A CP felt, “motivated…to participate in clinical trials,” after stating, “this study gave me some tools to ask researchers about getting results.” Many other participants expressed interest in research participation for themselves and in for members of their community. A CP began, “telling people that they should try to participate in more clinical studies because of how informational the readings are and that it is a great fulfilling experience.”

A PWP illustrated her interest in participating in research by saying, “For example, I want a cure, and I'm thinking, I never thought of looking into other research resources. You can't just stop at one page and not continue. I want to look into more and get in touch with researchers at Emory.” A PWP revealed that TeleDREAMS helped to, “refocus (her) into trying to prioritize what is the most important,” and to her, participating in a “clinical trial or study…is very important.” Some participants even seemed to understand the importance of research for future generations. One of these participants, a PWP noted, “Just participating in the trials might help my condition…but they are not designed to benefit me. They are designed to help the future.”

Across modules and participants, the two most popular themes were how knowledge diverged from the original intent of the module and understanding the importance of advocacy.

**Figure 5:**
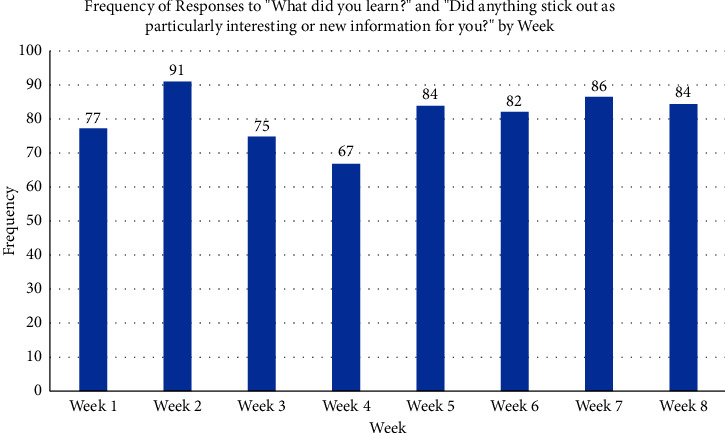
Frequency of participant responses to the questions “What did you learn?” and “Did anything stick out as particularly interesting or new information to you?,” that were specific to the topics and goals of the week. Data resulted from 716 participant responses over the 8-week program. Responses that did not fall under the weekly topic but reported new information learned (*n* = 70) were included in this total. See Table 2 for the topics that coincide with each week.

Figure 5 depicts the overall responses to the questions related to what was learned, and what was novel for the participant. The data show how different modules elicited more comments than others and depict the volume of overall responses to the questions. Research nuts and bolts (Module 2) got the most comments or responses to these questions, while interpreting the Clinical Trial got the fewest (Figure 5).

## 4. Discussion

### 4.1. General Findings

TeleDREAMS combined distance-learning and phone-based assessments to create an accessible approach that promotes research advocacy for older adults living with PD. TeleDREAMS also sought to increase participation among PWP and CP who are critically underrepresented in research. Information gathered from these phone calls is important in analyzing the needs, opinions, and attitudes of older adults towards research participation. In addition, the diverse demographic and clinical characteristics of the CP and PWP in this study may have shaped participants' experiences. The presence of comorbidities likely influenced participants' responses.

While TeleDREAMS appeared to increase interest in community engagement, research participation, and advocacy roles, participant responses to two specific questions “What did you learn?” and “Did anything stick out as particularly interesting or new information for you?” reveal that responses may have remained relatively constant across the eight weeks (Figure 5) with a peak at week 2 and a low at week 4.

### 4.2. Telehealth Educational Programs

Telehealth educational programs combined with phone-based assessments may be similar, or more effective, than traditional in-person methods of educational programs [16]. Telehealth education appeared as a promising alternative to face-to-face health promotion and programming during the 2003 severe acute respiratory syndrome outbreak in Hong Kong [17]. Therefore, TeleDREAMS may also have the potential to improve educational outreach programs to older adults during similar public health crises, such as the SARS-CoV-2 (COVID-19) pandemic. Caregivers of people with PD clearly had increased burden because of the coronavirus pandemic [18, 19].

### 4.3. Findings Related to Advocacy, Autonomy, Community Engagement, and Research Participation

The themes, “Understanding the Importance of Advocacy” (Theme 1) and “Becoming Cognizant of Past Advocacy Experiences” (Theme 2) are critical in considering how older adults view research advocacy and the roles and responsibilities of advocates. The purpose of TeleDREAMS was to provide a research advocacy program that educated older adults on the research process and role of participation in research; Themes 1 and 2 reveal that participants not only learned about research advocacy but were also able to apply advocacy to their lives. The findings of “Recognizing Patient Autonomy” overlap with our findings on advocacy, where some participants wanted to advocate for others' autonomy. An autonomous individual needs both intentional and full understanding of their actions [20], and both autonomy and advocacy require some degree of self-awareness and informed decision making [21]. More research is needed to understand the connection between patient advocacy and autonomy.

The identified themes “Community Engagement” (Theme 3) and “Research Participation” (Theme 8) which reveal that TeleDREAMS may have potential to improve older adults' participation in both research and community programs. These findings are consistent with the findings of the earlier DREAMS Program.

### 4.4. Findings Related to the Education of Older Adults

The most surprising finding of this study came from understanding the learning preferences of older adults. Participants seemed to enjoy learning from the personal stories and example-based lessons in their educational modules in Theme 5. This finding is consistent with previous research that advises information presented to older adults contain personal meaning [22, 23]. More research is needed to determine if a narrative format allows older adults to better understand traditionally difficult content.

Though each weekly module contained unique learning objectives, participants frequently reported learning about topics distinct from them. This observation was identified in “Knowledge Acquisition Diverges from the Module Intention” (Theme 6). Previous familiarity with information may have influenced how participants retained knowledge distinct from the module's intention [24]. During Week 6, a PWP, along with many other participants, claimed to have prior knowledge of health literacy and informed consent, but still expressed deeper moments of understanding after the module.

A recent study found that while healthy adults may be familiar with research subject protection measures, many are unfamiliar with informed consent, even when individuals have been asked to participate in research before [25]. That study, and supported by our findings in Theme 6, suggests that aspects of the research process remain unclear to many people.

### 4.5. TeleDREAMS Successes

As previously mentioned, many participants enjoyed learning from others who have experienced similar issues through personal stories. Most participants could relate the program's content to their own lives, and many found the content easy to share with others in their community.”

Overall, most participants appeared willing to participate in phone call interviews and some simply enjoyed the phone call conversations themselves. Weekly phone calls that coincided with each weekly module seem to be an effective method for holding participants accountable. Furthermore, dividing content into eight weekly modules may be beneficial for participants who have difficulty with cognitive functioning or completing tasks independently. In addition, accessible phone-based communication ensured participants had the opportunity to provide feedback with minimal technological concerns and did not have the burden of travel.

### 4.6. Areas of Improvement

During the phone calls, participants suggested methods of summarizing information and reorganizing the structure within each module.

A CP's spouse suggested using an index or glossary at the end of each module. Summarizing and organizing each weekly module through different methods may help participants feel less “overloaded” with information.

Even with conscious efforts to make the educational content more accessible, some participants still struggled with what they called, “terminology and nomenclature,” or “technical stuff.” A CP said, “the jargon was kinda confusing and unnecessary,” for their care recipient to understand. In addition to organizing each module and highlighting key terms, adjusting the content from an eighth-grade reading level to a fifth-grade reading level may allow print material to be understood by more participants [26].

Several participants requested more information about PD specifically. Participants were interested in resources related to PD research, symptom management, diagnoses, and preventative measures. This finding aligns with previous research that suggests adult learners are driven by self-autonomy and emotional and physical limitations [23]. Lastly, participants requested more examples, personal stories, and testimonials, thus emphasizing our observation that they benefitted from, and enjoyed example-based learning.

## 5. Limitations

We acknowledge some important limitations. Approximately 1/5 of all PD cases in the United States are diagnosed in non-Caucasian groups. While PWP in this study reflects possibly greater than national PD incidence (28.1% PWP from non-White groups), work is still necessary to recruit more participants from underserved backgrounds. Further, this study's findings are only generalizable to the historically disadvantages groups that make up the study's sample. Future recruitment strategies should target individuals from all racial and ethnic minority groups, those from lower socioeconomic status, and lower education to ensure that research findings reflect the diversity in our communities [1].

In addition, this analysis was limited by the inability to track theme evolution over time. Our findings suggest that participants increased interest in community engagement, research participation, and advocacy roles, but we are unable to determine when participants' views began to change. We do not have the breakdown of theme iteration for specific modules. This study was not designed to examine the differences and variability within the CP or PwP groups; however, these differences are important and should be evaluated in future research.

Another limitation of this study was that weekly telephone interviews were neither transcribed nor recorded. Without proper transcriptions, participants' responses may be incomplete. The field notes were also completed by different research staff who documented the calls in slightly varied formats. Reworkings of TeleDREAMS will greatly benefit from recording and transcribing telephone interviews.

## 6. Conclusion

The feedback from participants, along with an expressed desire to participate in research and community programs suggests research advocacy training enhances research participation and general understanding and outlook of the research process of older adults, a finding aligning with previous studies including in other populations [27]. Based on these findings, both CP and PwP appreciated and may need to interact and communicate with others about topics germane to clinical research to feel a part of the ever changing and growing community of Parkinson's. Factors related to diversity and differences between groups are also important to discuss and process with these groups of individuals [28]. The combined model of distance-learning and phone-based assessment is an accessible means of interacting with hard-to-reach participants. Future iterations of TeleDREAMS may benefit from incorporating ample personal stories, clearly identified terminology, and well-summarized modules. This qualitative model and its results are useful for designing telehealth educational programs such as TeleDREAMS.

## Figures and Tables

**Table 1:** Demographic characteristics of PWP versus the CP groups.

	Total	PWP	CP
*N*	51	32 (62.75)	19 (37.25)
Age (year)	67.55 ± 7.6	68.06 ± 8.3	66.68 ± 6.4
Education (year)	16.33 ± 2.5	16.06 ± 2.8	16.82 ± 1.7
BMI	27.25 ± 6.2	26.45 ± 5.8	28.61 ± 6.8
Composite physical function	19.74 ± 5.4	17.88 ± 5.9	23.06 ± 1.4

*Sex*			
Men	28 (54.9)	22 (68.6)	6 (31.6)
Women	23 (45.1)	10 (31.2)	13 (68.4)

*Race*			
Asian	3 (5.9)	1 (3.1)	2 (10.5)
Black	11 (21.6)	7 (21.9)	4 (21.1)
Hispanic or Latino	2 (3.9)	1 (3.1)	1 (5.3)
White	35 (68.6)	23 (71.9)	12 (63.2)

*House type*			
House/apartment/condominium	49 (96.1)		
Senior housing (independent)	2 (3.9)		

*Leave house*			
1-2 times/week	8 (16)	7 (21.9)	1 (5.6)
3-4 times/week	12 (240)	8 (25)	4 (22.2)
Every day	29 (58)	16 (50)	13 (72.2)
Less than once/week	1 (2)	1 (3.1)	0 (0)

*Use of assistive device*			
No	35 (70)	17 (53.1)	18 (100)
Sometimes	8 (16)	8 (25)	0 (0)
Yes	7 (14)	7 (21.9)	0 (0)

**Table 2:** Module topics in TeleDREAMS educational content.

Week	Topic
1	Introduction to Research Advocacy
2	Research Nuts and Bolts
3	Ethics and Aging Research
4	Understanding and Interpreting Clinical Trials for Patient Advocates
5	Aging and Clinical Research
6	Informed consent, Understanding the Issues and Health Literacy
7	Effective Advocacy in the Clinical Research Process
8	Engaging Diverse Communities in Research and Getting Started as a Research Advocate

## Data Availability

The data used to support the findings of this study are available from the corresponding author upon reasonable request.

## Appendix

  Questions asked by study staff to participants during weekly phone call interviews
(1) Have you read the Week <1, 2, 3, etc.> lesson?
(2) Did you look at any extra information about this topic, such as websites/videos/supplemental materials?
(3) What did you learn?
(4) Did anything stick out as particularly interesting or new information for you?
(5) What did you know about (topic) before reading this lesson?
(6) Did you learn anything that you can use in your own life?
(7) Was there anything else related to (topic) that you think should have been included in this week's module?
(8) Do you have any other comments about this week's module or topic?
